# Mast cells participate in regulation of lung‐gut axis during *Staphylococcus aureus* pneumonia

**DOI:** 10.1111/cpr.12565

**Published:** 2019-02-07

**Authors:** Chao Liu, Liping Yang, Yu Han, Wei Ouyang, Wei Yin, Feng Xu

**Affiliations:** ^1^ Department of Infectious Diseases The Second Affiliated Hospital Zhejiang University School of Medicine Hangzhou China; ^2^ Core Facilities Zhejiang University School of Medicine Hangzhou China

**Keywords:** intestinal microbiota, lung‐gut axis, mast cells, *Staphylococcus aureus* pneumonia

## Abstract

**Objectives:**

The lung‐gut axis is known to be involved in the pathogenesis of *Staphylococcus aureus* pneumonia. However, the underlying mechanisms remain unclear. We examined the role of pulmonary mast cells (MCs) in the regulation of the lung‐gut axis during *S. aureus* pneumonia.

**Materials and Methods:**

We created a mouse model of *S. aureus* pneumonia using MC‐deficient mice (*Kit* ^*W‐sh/W‐sh*^) and examined the level of inflammation, bacterial burden, expression of cathelicidin‐related antimicrobial peptide (CRAMP) and composition of the gut microbiota. We further evaluated anti‐bacterial immunity by administering bone marrow MCs (BMMCs) or CRAMP into the lungs of *Kit* ^*W‐sh/W‐sh*^ mice.

**Results:**

After *S. aureus* challenge, the MC‐deficient mice, compared with wild‐type (WT) mice, displayed attenuated lung inflammation, decreased expression of CRAMP, higher bacterial lung load and disturbance of the intestinal microbiota. Adoptive transfer of BMMCs into the lung effectively reconstituted the host defence against *S. aureus* in *Kit* ^*W‐sh/W‐sh*^ mice, thus resulting in recovery of *S. aureus* pneumonia‐induced intestinal dysfunction. Similarly, exogenous administration of CRAMP significantly enhanced anti‐bacterial immunity in the lungs of MC‐deficient mice.

**Conclusions:**

This study provides evidence for the involvement of MCs in the regulation of the lung‐gut axis during *S. aureus* pneumonia.

## 1 INTRODUCTION

*Staphylococcus aureus* is one of the common pathogens in hospital‐ and community‐acquired pneumonia.^1^ In addition to the symptoms including headache, fever, dyspnoea and cough, patients with pneumonia often present with gastroenteritis‐like discomforts such as vomiting and diarrhoea, especially in young children.^2^ Increasing evidence indicates that the intestinal microbiome and mucosal tissues broadly affect the progression of multiple diseases.^3^ The lung‐gut axis, which represents the interaction between lung immunity and intestinal microbiota,^4^ is thus an area of interest, though the underlying mechanisms remain poorly understood.

Mast cells (MCs) are particularly abundant at host‐environment interfaces, such as the skin and the respiratory and gastrointestinal tracts. Because of their location, MCs have been hypothesized to act as sentinel cells that sense pathogen attacks and initiate a protective immune response.^5^ However, knowledge of the role of MCs in the host defence against *S. aureus* is limited. Recent studies have demonstrated that MCs can be activated by *S. aureus* and exert antimicrobial activity.^6^, ^7^ But, anti‐*S. aureus* mechanisms of MCs have not yet been demonstrated in the lung. The lung is the major target tissue of diverse inhaled microbial pathogens, including *S. aureus*. To combat them, the respiratory system is armed with diverse mechanisms of innate mucosal immunity, including the secretion of antimicrobial peptides.^8^, ^9^ Cathelicidins show anti‐bacterial activity against both Gram‐positive and Gram‐negative bacteria through destruction of bacterial membrane.^10^, ^11^, ^12^ Cathelicidin expression is upregulated in the airways during bacterial infection^13^ and has been detected in alveolar macrophages, neutrophils and airway epithelial cells.^10^, ^14^, ^15^ There has been increasing interest in the role of cathelicidin‐related antimicrobial peptide (CRAMP) during bacterial pneumonia.

C‐kit receptor plays crucial roles in the differentiation, proliferation and inflammatory reaction of MCs.^16^, ^17^ Our prior work has shown that MCs released inflammatory factors (eg, tumour necrosis factor (TNF)‐α) and CRAMP in response to *S. aureus*, through stimulation of c‐kit receptor and its downstream molecules.^18^ In this study, using MC‐deficient mice (*Kit* ^*W‐sh/W‐sh*^) with defective c‐kit receptor, we evaluated the diversity of the intestinal microbial community in mice with *S. aureus* pneumonia and determined the role of MCs in regulation of the lung‐gut axis. We showed that MC deficiency impaired lung inflammation and aggravated the imbalance in the gut microbiota upon *S. aureus* infection. Adoptive transfer of bone marrow mast cells (BMMCs) into the lung largely reconstituted the host defence against *S. aureus* in both the lung and the gut, thus demonstrating a critical role of MCs in the immune response through regulation of the lung‐gut axis.

## 2 MATERIALS AND METHODS

### 2.1 Animals

*Kit* ^*W‐sh/W‐sh*^ mice on a C57BL/6 background were kindly provided by Harvard Medical School.^19^ Age‐ and sex‐matched wild‐type (WT) mice were used as controls. All animal experiments were approved by the Animal Care and Use Committee of The Second Affiliated Hospital, Zhejiang University School of Medicine.

### 2.2 Bacterial culture

A clinical isolate of *S. aureus* was grown aerobically overnight at 37°C in a shaking incubator.^18^, ^20^ *S. aureus* in the mid‐logarithmic phase was resuspended in phosphate‐buffered saline (PBS). The numbers of bacteria was quantified according to the OD600‐based bacterial growth curve and verified with colony‐forming unit (CFU) assays.^20^

### 2.3 BMMCs culture

Bone marrow mast cells were generated from the femurs of C57BL/6 mice and maintained in the presence of 10% pokeweed mitogen‐stimulated spleen‐conditioned medium, as described previously.^18^, ^21^ After 4 weeks of culture, about 99% of the cells had developed into MCs.^21^

### 2.4 A mouse model of *S. aureus* lung infection

Eight‐week‐old C57BL/6 and *Kit* ^*W‐sh/W‐sh*^ mice were used to establish an *S. aureus* pneumonia model.^22^ In brief, 40 μL of *S. aureus* (5 × 10^7^ CFUs) was inoculated intratracheally into anesthetized mice. Non‐infected control mice were administered with an equal volume of PBS intratracheally. Lung specimens were weighed and homogenized in 1 mL PBS to determine bacterial load and production of cytokines and CRAMP.

### 2.5 Reconstitution of MCs in *Kit* ^*W‐sh/W‐sh*^ mice

*Kit* ^*W‐sh/W‐sh*^ mice (5‐6 weeks old) were injected with 1 × 10^7^ BMMCs in 200 μL PBS via the tail vein. The mice treated with PBS were used as controls. After 3 weeks, the reconstituted mice were used to build *S. aureus* pneumonia model, and tissues were harvested for analysis after 24 hour post‐infection.^23^

### 2.6 CRAMP treatment

The effects of CRAMP on *S. aureus* pneumonia were examined through intratracheal administration of 20 mg/kg CRAMP (GL Biotech, Shanghai, China) in 40 μL PBS to mice for four consecutive days.^24^

### 2.7 ELISA

The levels of cytokines including interleukin (IL)‐1β, IL‐6, keratinocyte‐derived chemokine (KC), macrophage inflammatory protein (MIP)‐2, TNF‐α and CRAMP in lung homogenates were detected using enzyme‐linked immunosorbent assay (ELISA) kits according to the manufacturer's instructions.

### 2.8 Histological analysis

Mouse lung specimens were fixed in 10% paraformaldehyde, embedded in paraffin, cut into 4 μm‐thick sections and stained with haematoxylin‐eosin (H&E). The stained sections were reviewed for morphology under a photomicroscope (Leica, Heidelberg, Germany). The neutrophil infiltration and inflammatory scores of the lung samples were determined as described previously.^25^, ^26^ In brief, for evaluation of lung neutrophils, three lung sections from each mouse were analysed and three randomly selected high‐power fields (HPF) were examined for each lung section, the average value of cells/HPF for each mouse was determined by summation of all numbers divided by 9. The inflammatory levels were semi‐quantitatively determined as below: peribronchial/peribronchiolar inflammation 0‐4, perivascular inflammation 0‐4 and alveolar inflammation 0‐2. Each lung section was given a total inflammatory score (maximum score of 10).

### 2.9 Immunofluorescence staining

Paraffin‐wax sections of mouse lung tissues were placed on glass slides, and non‐specific antibody binding was blocked by incubation with 1% bovine serum albumin for 1 hour. Alexa Fluor 488 anti‐mouse CD117 (c‐kit) antibody (1: 50, BioLegend, San Diego, CA, USA) and phycoerythrin anti‐mouse CRAMP antibody (1: 50, BioLegend) were mixed and used for double staining. The sections were incubated with the above antibodies overnight at 4°C, then incubated at 37°C for 30 minutes. DAPI was used to stain the nuclei before viewing by confocal microscopy (Olympus, Tokyo, Japan).

### 2.10 Analysis of the faecal microbiota

Faecal samples in ileocecus were collected from WT and *Kit* ^*W‐sh/W‐sh*^ mice 24 hours after *S. aureus* infection, then snap‐frozen in liquid nitrogen. Faecal DNA was extracted and used for 16S rRNA sequencing according to manufacturer's instructions (Tianke, Hangzhou, China). Total genomic DNA was extracted using the PowerSoil^®^ DNA Isolation Kit. 16S rRNA/ITS genes of distinct regions were amplified. PCRs were carried out with Phusion^®^ High‐Fidelity PCR Master Mix. PCR products were mixed in equidensity ratios and purified with Qiagen Gel Extraction Kit. Sequencing libraries were generated using TruSeq^®^ DNA PCR‐Free Sample Preparation Kit and examined on an Illumina MiSeq platform.

### 2.11 Statistical analysis

Data are expressed as mean ± standard error of mean (SEM), unless otherwise stated. Differences between two or multiple groups were analysed with Student's *t* test or one‐way ANOVA as appropriate. CFUs in lung tissue were compared using the Mann–Whitney *U* test. A *P* value <0.05 was considered statistically significant. All calculations were performed in GraphPad software (Version 5.01).

## 3 RESULTS

### 3.1 MC deficiency decreases the inflammatory response to *S. aureus* infection

**Figure 1:**
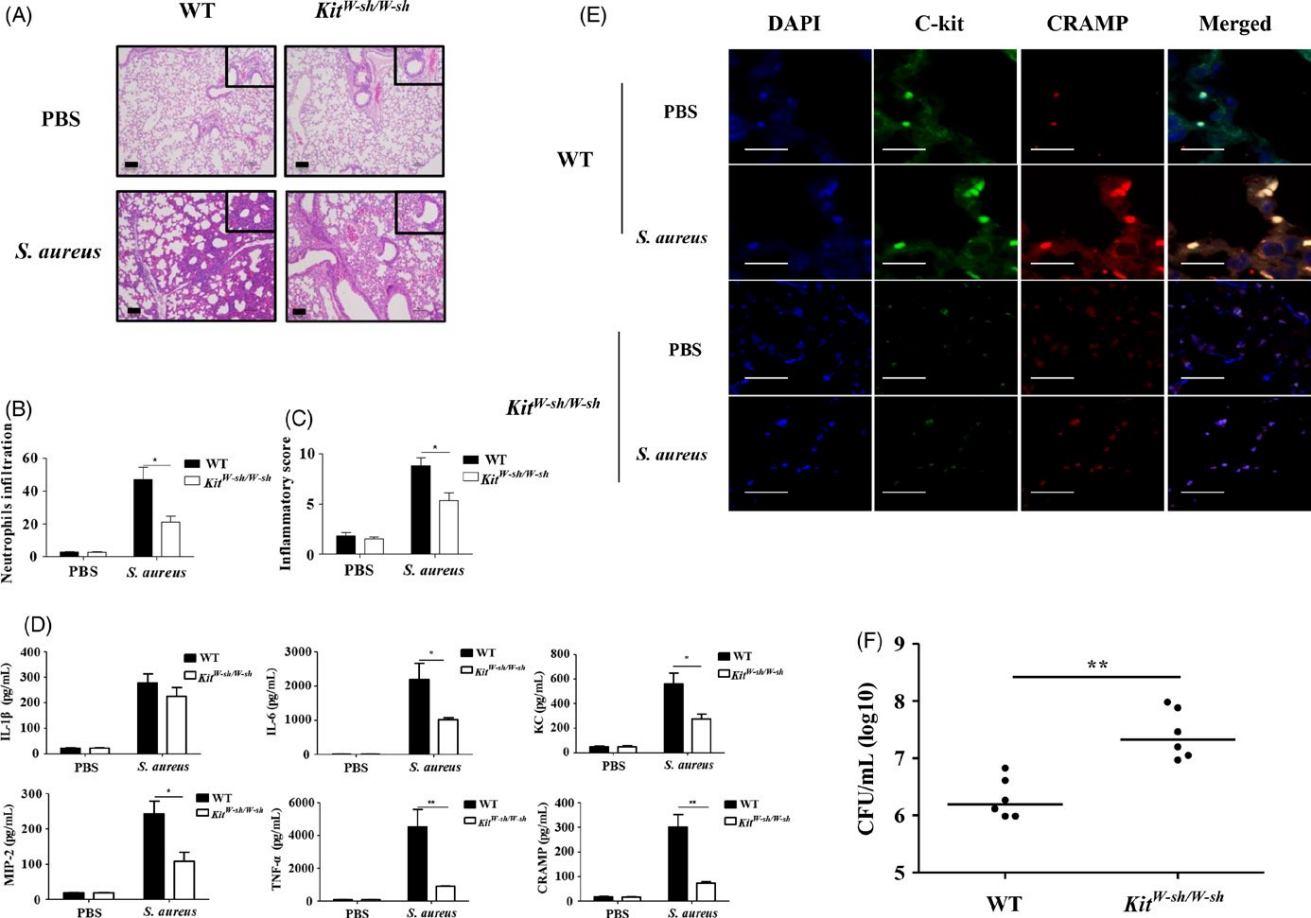
*Kit* ^*W‐sh/W‐sh*^ mice are susceptible to *Staphylococcus aureus* infection. WT and *Kit* ^*W‐sh/W‐sh*^ mice (n = 6 per group) were injected intratracheally with PBS or *S. aureus*. Lung tissues were harvested at 24 hours after infection. (A) Representative H&E‐stained lung‐tissue sections. Magnification 400× for the inserts, 100× for all others; scale bar, 100 μm. (B) Evaluations for lung neutrophils. (C) Inflammatory scores. (D) Levels of IL‐1β, IL‐6, KC, MIP‐2, TNF‐α and CRAMP. (E) CRAMP expression in c‐kit^+^ lung MCs by immunofluorescence staining. Magnification 180×; scale bar, 20 μm. (F) Bacterial load in lungs. Individual values and medians are shown for each group. *P <* *0.05 and **P <* *0.01

We evaluated the effect of MCs on the inflammatory response to *S. aureus* infection. There were no differences in lung neutrophil counts between uninfected *Kit* ^*W‐sh/W‐sh*^ mice and WT animals; however, the inflammatory levels in *Kit* ^*W‐sh/W‐sh*^ mice were significantly lower than those in WT mice, based on H&E staining at 24 hours after infection (Figure 1A‐C). We further detected the levels of inflammatory cytokines including IL‐1β, IL‐6, KC, MIP‐2, TNF‐α and CRAMP. The expression levels of all cytokines except IL‐1β were significantly lower in lung specimens from *Kit* ^*W‐sh/W‐sh*^ mice than those from WT mice 24 hours post‐infection (Figure 1D). Confocal microscopy examinations showed that *S. aureus* stimulation upregulated the expression of lung MC‐derived CRAMP in WT mice, but weak expression of CRAMP was found in *Kit* ^*W‐sh/W‐sh*^ mice (Figure 1E). Meanwhile, *Kit* ^*W‐sh/W‐sh*^ mice had significantly higher numbers of *S. aureus* in their lungs after infection, compared to WT mice (Figure 1F).

### 3.2 *Kit* ^*W‐sh/W‐sh*^ mice display impaired intestinal microbiota after *S. aureus* lung infection

**Figure 2:**
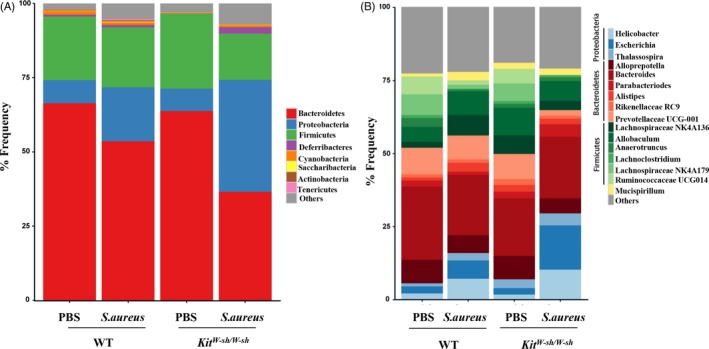
MCs alter the faecal microbiota composition after *Staphylococcus aureus* infection. Faecal samples were collected from WT and *Kit* ^*W‐sh/W‐sh*^ mice (n = 5 per group) at 24 h after infection. Analysis of faecal microbiota in WT and *Kit* ^*W‐sh/W‐sh*^ mice was performed by 16S rRNA sequencing. Composition of the intestinal microbiota was showed at the phylum level (A) and genus level (B), respectively

To explore the influence of MCs on the intestinal microbiota, we analysed the intestinal microbiota using 16S rRNA sequencing at 24 hours post‐infection. The mouse intestinal microbiota consisted of three major bacterial phyla: *Proteobacteria*,* Bacteroidetes* and *Firmicutes*. Abundant *Proteobacteria* were found in the intestinal microbiota in the infected mice. *Kit* ^*W‐sh/W‐sh*^ mice showed higher burden of *Proteobacteria* than WT mice after pulmonary *S. aureus* infection (Figure 2A). *E. coli* is an important component of the *Enterobacteriaceae* and a common cause of vomiting and diarrhoea in humans.^27^ Notably, the most striking change in the faecal microbial community after *S. aureus* infection was an increase in the abundance of the genus *Escherichia* (Figure 2B), which accounted for approximately 2% of the total faecal microbiota in uninfected WT and *Kit* ^*W‐sh/W‐sh*^ mice, but raised to approximately 6% and 15%, respectively, in infected mice.

### 3.3 Adoptive transfer of BMMCs reconstitutes host defence against *S. aureus* infection in *Kit* ^*W‐sh/W‐sh*^ mice

**Figure 3:**
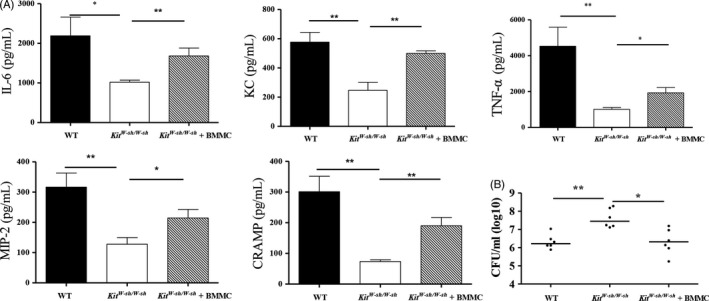
BMMCs transfer reconstitutes host resistance to *Staphylococcus aureus* infection in *Kit* ^*W‐sh/W‐sh*^ mice. WT,* Kit* ^*W‐sh/W‐sh*^ and reconstituted *Kit* ^*W‐sh/W‐sh*^ mice (n = 6 per group) were injected intratracheally with *S. aureus*. Lung tissues were collected at 24 h after infection. (A) Levels of IL‐6, KC, MIP‐2, TNF‐α and CRAMP in lung tissues. (B) *S. aureus* counts in lung tissues. Individual values and medians are shown for each group. *P <* *0.05 and **P <* *0.01

**Figure 4:**
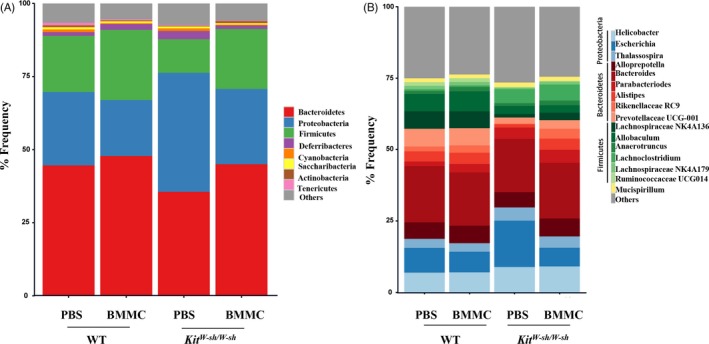
Intestinal microbiota is reversed in MC‐reconstituted *Kit* ^*W‐sh/W‐sh*^ mice. Faecal samples were collected from WT, reconstituted WT, * Kit* ^*W‐sh/W‐sh*^ and reconstituted *Kit* ^*W‐sh/W‐sh*^ mice (n = 5 per group) at 24 h after infection. Analysis of faecal microbiota was performed by 16S rRNA sequencing. Composition of the intestinal microbiota was shown at the phylum level (A) and genus level (B), respectively

We performed an adoptive MC transfer experiment to determine whether the susceptibility of *Kit* ^*W‐sh/W‐sh*^ mice to *S. aureus* was mainly attributed to MC deficiency. Treatment of WT‐derived BMMCs led to significantly elevated levels of IL‐6, TNF‐α, KC, MIP‐2 and CRAMP in *KitW-sh/W-sh* mice upon infection (Figure 3A). Correspondingly, MC‐reconstituted *Kit* ^*W‐sh/W‐sh*^ mice exhibited markedly improved bacterial clearance of *S. aureus* (Figure 3B). Furthermore, sequencing analysis of the microbiota also indicated that the imbalance in the intestinal flora after *S. aureus* infection was largely reversed in MC‐reconstituted *Kit* ^*W‐sh/W‐sh*^ mice (Figure 4A‐B).

### 3.4 Exogenous CRAMP treatment significantly ameliorates *S. aureus* pneumonia

**Figure 5:**
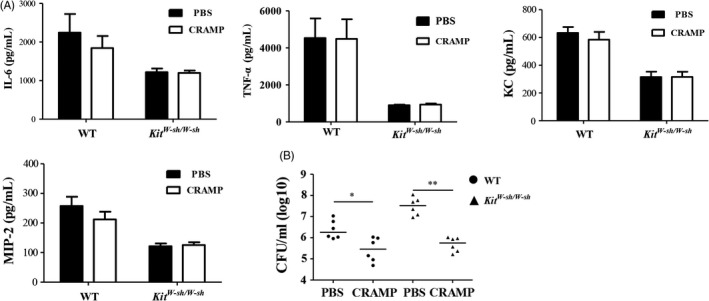
Treatment with CRAMP restores the host defence against *Staphylococcus aureus* infection in *Kit* ^*W‐sh/W‐sh*^ mice. WT and *Kit* ^*W‐sh/W‐sh*^ mice (n = 6 per group) were intratracheally injected with mouse CRAMP (20 mg/kg) or PBS for four consecutive days prior to *S. aureus* infection (5 × 10^7^ CFUs). Lung tissues were harvested at 24 h after infection. (A) Levels of IL‐6, KC, MIP‐2 and TNF‐α in lung tissues. (B) *S. aureus* counts in lung tissues. Individual values and medians are shown for each group.*P <* *0.05 and **P <* *0.01

Exogenous CRAMP was intratracheally administrated into the mice for 4 consecutive days. Although the lung‐tissue levels of the pro‐inflammatory cytokines IL‐6, TNF‐α, KC and MIP‐2 were not significantly affected by CRAMP treatment (Figure 5A), the bacterial load was obviously decreased by CRAMP treatment in both WT and *Kit* ^*W‐sh/W‐sh*^ mice (Figure 5B).

## 4 DISCUSSION

*Staphylococcus aureus* is one of the most common causative pathogens of pneumonia.^28^ Although pneumonia is known to be a lung infection, many studies have shown that pneumonia is also associated with substantial extrapulmonary effects, including intestinal inflammation, apoptosis and injury.^29^, ^30^ Mucosal tissues, including those in the respiratory and gastrointestinal tracts, act as the first line of host defence against invading pathogens. Gill et al^31^ have proposed that the mucosal immune system, which is associated with the commensal microbial community, is not isolated. Agwu et al^32^ observed that respiratory *S. aureus* infection in mice caused immune injury not only in the lung but also in the gut, and resembled the symptoms exhibited by humans infected with *S. aureus*. *Staphylococcus aureus*‐infected mice provide a good model for exploring the mechanisms of lung‐gut axis during respiratory *S. aureus* infection. Furthermore, these observations give more evidence supporting the existence of a broad mucosal immune system.

Mast cells act as effector cells during infection and are increasingly recognized to have a major role in modulating the course of infection.^33^ Previous studies demonstrated that *S. aureus* infection causes a series of inflammatory responses in the lung, in line with infiltration of various inflammatory cells including neutrophils, macrophages and natural killer cells.^34^, ^35^ Nevertheless, the role of MCs in host defence against *S. aureus* infection is largely unknown. *Kit* ^*W‐sh/W‐sh*^ mice with the *Kit* ^*W‐sh*^ mutation were reported to have a significant deficiency of MCs in all tissues but normal levels in other differentiated hematopoietic and lymphoid cells, showing the dampen immune responses.^36^, ^37^ In this study, using *Kit* ^*W‐sh/W‐sh*^ mice, we revealed that MC‐deficient mice were significantly more susceptible to *S. aureus* lung infection than WT animals, accompanied by a greater imbalance of the intestinal flora. We further carried out adoptive transfer of BMMCs into *Kit* ^*W‐sh/W‐sh*^ mice prior to *S. aureus* infection, to obtain additional supportive evidence for the role of MCs in keeping intestinal microflora homeostasis. Interestingly, the suppressed inflammatory response was largely reconstituted in *Kit* ^*W‐sh/W‐sh*^ mice after adoptive BMMCs administration. Moreover, the *Kit^W-sh/W-sh^* mice received WT‐derived BMMCs had significantly fewer harmful bacteria in the gut than did controls. The present study thus indicates the critical role of MCs in keeping the host defence and maintaining gut microbial homeostasis against *S. aureus* infection.

Inflammation is an integral part of the reactions of the innate immune systems against micro‐organisms.^38^ We recently showed that the activated MCs contributed to a series of inflammatory actions to clear the pathogen in a mouse model of skin *S. aureus* infection.^18^ The host inflammatory and antimicrobial responses against *S. aureus* were impaired in *Kit* ^*W‐sh/W‐sh*^ mice, which suggested that MCs may act as an immune booster in the lung. Consistently, our previous data showed that MCs released multiple inflammatory cytokines directly upon *S. aureus* infection by means of the c‐kit‐activated phosphoinositide 3‐kinase (PI3K)/AKT/P65‐nuclear factor (NF‐κB) signal pathway.^18^

A couple of studies have revealed that MCs can inhibit bacterial growth by secreting the murine cathelicidin CRAMP.^39^, ^40^ CRAMP expression in the lung was significantly upregulated in response to different pathogen invasion.^9^, ^12^, ^41^ Here, we reported that CRAMP expression in the lung increased after infection, directly proportional to the amounts of MCs. Moreover, exogenous CRAMP treatment effectively decreased bacterial burden in a model of *S. aureus* pneumonia.

In summary, our results demonstrated that lung MCs are involved in regulation of the lung‐gut axis during *S. aureus* pneumonia. This study thus provides a potential clinical strategy focused on MCs for patients with *S. aureus* pneumonia accompanied by severe gut symptoms.

## CONFLICT OF INTEREST

The authors declare no conflict of interest.
